# Risk Factors for Visceral Leishmaniasis among Residents and Migrants in Kafta-Humera, Ethiopia

**DOI:** 10.1371/journal.pntd.0002543

**Published:** 2013-11-07

**Authors:** Daniel Argaw, Abate Mulugeta, Mercè Herrero, Nohelly Nombela, Tsegemariam Teklu, Teodros Tefera, Zewdu Belew, Jorge Alvar, Caryn Bern

**Affiliations:** 1 Department for the Control of Neglected Tropical Diseases (CDS/NTD/IDM), Leishmaniasis Control Program, World Health Organization, Geneva, Switzerland; 2 Disease Prevention and Control Programmes, World Health Organization, Addis Ababa, Ethiopia; 3 Tigray Regional Health Office, Mekelle, Tigray Regional State, Ethiopia; 4 Kashay Abera Hospital, Kafta-Humera, Tigray Regional State, Ethiopia; 5 Department of Epidemiology and Biostatistics, University of California San Francisco, San Francisco, California, United States of America; University of Pittsburgh, United States of America

## Abstract

**Background:**

Visceral leishmaniasis is a lethal parasitic disease transmitted by phlebotomine sand flies. The largest focus of VL in Ethiopia is located in the lowland region bordering Sudan, where the epidemiology is complicated by the presence of thousands of seasonal agricultural workers who live under precarious conditions.

**Methodology/Principal Findings:**

We conducted two parallel case-control studies to identify factors associated with VL risk in residents and migrants. The studies were conducted from 2009 to 2011 and included 151 resident cases and 157 migrant cases, with 2 matched controls per case. In multivariable conditional regression models, sleeping under an acacia tree at night (odds ratios (OR) 5.2 [95% confidence interval 1.7–16.4] for residents and 4.7 [1.9–12.0] for migrants), indicators of poverty and lower educational status were associated with increased risk in both populations. Strong protective effects were observed for bed net use (OR 0.24 [0.12–0.48] for net use in the rainy season among residents, OR 0.20 [0.10–0.42] for any net use among migrants). For residents, living in a house with thatch walls conferred 5-fold and sleeping on the ground 3-fold increased risk. Among migrants, the risk associated with HIV status was borderline significant and sleeping near dogs was associated with 7-fold increased risk.

**Conclusions/Significance:**

Preventive strategies should focus on ways to ensure net usage, especially among migrant workers without fixed shelters. More research is needed to understand migration patterns of seasonal labourers and vector bionomics.

## Author Summary

Visceral leishmaniasis is a lethal parasitic disease transmitted by sand flies. The largest focus of VL in Ethiopia is located in the lowland region bordering Sudan, where hundreds of thousands of agricultural workers migrate for work every year during the planting and harvest seasons. We conducted two parallel studies in residents and migrants to determine the living conditions and behaviors that put people at higher risk of VL risk. We found that sleeping under an acacia tree at night, indicators of poverty and lower educational status were associated with increased risk in both populations. Sleeping under a bed net was protective. For residents, living in a house with thatch walls and sleeping on the ground increased risk of VL. Among migrants, the risk associated with HIV status was borderline significant and sleeping near dogs was associated with increased risk. Preventive strategies should focus on ways to ensure net usage, especially among migrant workers without fixed shelters. More research is needed to understand migration patterns of seasonal labourers and vector behavior.

## Introduction

Visceral leishmaniasis (VL) is a lethal parasitic disease transmitted by phlebotomine sand flies. The typical clinical picture is a chronic systemic illness with fever, weight loss, splenomegaly, hepatomegaly, and bone marrow suppression with pancytopenia. East Africa is second only to the Indian subcontinent in annual VL incidence [1]. Although most transmission is thought to be anthroponotic, *Leishmania donovani* has also been found in dogs and wild mammals in Sudan and Ethiopia [2]–[4]. The risk of acquiring the disease is mediated through poor housing conditions, lack of personal protective measures against the vector and economically-driven migration that brings non-immune hosts into VL-endemic areas [5].

In 2005, a new VL epidemic was reported in Libo Kemkem in the highlands of north-western Ethiopia, where only a handful of cases had been reported in the past [2], [6]. The parasite was thought to have been introduced by agricultural labourers returning from seasonal work in the Humera-Metema lowland area bordering Sudan, a hypothesis supported by the high prevalence of positive leishmanin skin tests found in returned migrant workers and molecular tracking of strains from the two regions [2], [7]. The lowland border area hosts the largest VL focus in Ethiopia and is adjacent to VL-endemic areas of Sudan [5]. The main vector on both sides of the border is *Phlebotomus orientalis*, found in association with cracked black cotton-clay soils and acacia-balanites forest [8], [9]. Hundreds of thousands of seasonal agricultural workers migrate into the Humera-Metema area each year, staying months to years and often living under precarious conditions, and VL occurs in both long-term residents and migrants [10], [11]. In this complex epidemiological setting, understanding risk factors for VL is crucial for the design of appropriate interventions. We conducted two parallel case-control studies to identify factors associated with VL risk in residents and migrants.

## Methods

### Study area

**Figure 1:**
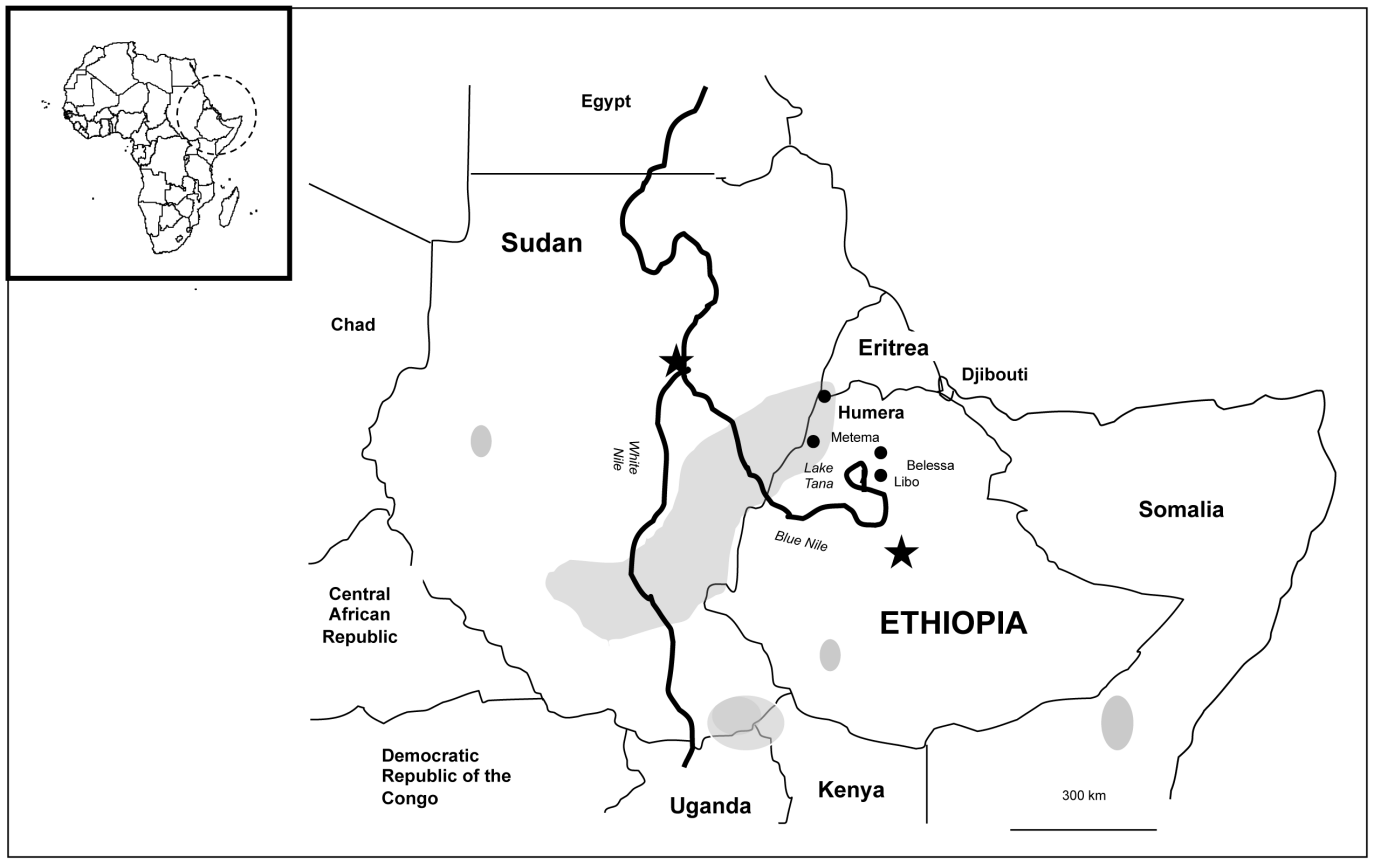
The major foci of kala-azar in the Horn of Africa, indicating the location of the study site of Humera near the borders with Sudan and Eritrea.

The studies were conducted in Kafta-Humera district, Tigray regional state, bordered on the west by Sudan, and on the north by the Tekezé River which separates Ethiopia from Eritrea (Figure 1). Kafta-Humera had an estimated total population of 92,144 in 2007; 32.8% were urban dwellers. The reported HIV prevalence in the district was 4.6% in 2009. Before the 1970s, the region reported only sporadic cases of VL. The Kafta-Humera district is one of Ethiopia's most fertile agricultural zones with large scale of farming of cash crops such as sesame, maize, cotton and sorghum. Since 1970, linked to an extensive program of agricultural development and the consequent increase in the influx of migrant workers, the number of VL cases has rapidly increased. Migrants travel to Kafta-Humera to work in the sowing and harvest seasons, mainly from October to February and May to July. Seasonal agricultural work attracts approximately 250,000 migrant laborers each year from non-endemic parts of Tigray and neighboring regions.

### Ethics statement

The study protocols were approved by the ethical review committees of the Ethiopian Public Health Association (resident protocol) and the Tigray Regional State Health Bureau (migrant protocol). Each adult participant provided written informed consent. A parent or guardian provided consent on behalf of child participants. All research activities adhered to the principles expressed in the Declaration of Helsinki. Approval to record existing hospital data on HIV status was given only for the migrant protocol; these data are therefore missing for the resident protocol.

### Study populations and periods

Because the resident and migrant populations differed substantially in terms of practical options for valid control selection and some aspects of exposure history, we performed the two investigations independently. An effort was made to maintain key variables in both investigations, but the difference in living circumstances required some questions to be worded differently.

Cases were ascertained for both studies at the Kashay Aberra Hospital (KAH) which contained a VL treatment center operated by *Médecins Sans Frontières* (MSF)-Holland until to June 2009. After June 2009, responsibility for VL treatment was assumed by regular hospital staff. This hospital is the main VL treatment facility for the area and treated 348 VL cases in 2009, 505 in 2010 and 405 in 2011. For both studies, VL case definitions followed the hospital diagnostic algorithm. A past VL case was a patient with at least 2 weeks of fever plus weight loss and/or splenomegaly, diagnosed by KAH based on positive DAT (antigen from the Royal Tropical Institute, Amsterdam, The Netherlands) or rK39 dipstick and treated with antileishmanial drug with clinical resolution of symptoms; or a patient with *Leishmania* amastigotes demonstrated in splenic aspirate. A current VL case was a patient diagnosed with illness characterized by at least 2 weeks of fever plus weight loss and/or splenomegaly, plus *Leishmania* amastigotes in the relevant aspirate and/or positive DAT or rK39 dipstick result. Both studies included 2 controls per case matched by sex and age group (<5, 5–14, 15–39 and ≥40 years). A meta-analysis estimated rK39 and DAT sensitivities of 79% and 93% in East African studies, with specificities of 85% and 96% respectively [12]. A recent article from the study hospital showed rK39 rapid test and DAT sensitivities of 84% and 94%, respectively [13]. Treatment was administered by the hospital staff following standard MSF and Ethiopian national treatment guidelines; in general, first-line treatment for non-severe VL consisted of 30 days of sodium stibogluconate 20 mg/kg/day whereas severely ill VL patients, relapses and those with HIV co-infection were treated with either conventional amphotericin B 1 mg/kg/day on alternate days for 30 days or AmBisome 5 mg/kg in 4 to 6 on alternate days to reach a total dose of 20 to 30 mg/kg. Treatment and outcomes at this hospital were described in an earlier publication [14].

We used a community-based strategy to recruit controls for the resident study. Because a community-based approach was impossible for the migrant study due to the transience of the migrants, we recruited controls for this study in the hospital on services other than the VL treatment service. The two studies were therefore conducted independently, although an effort was made to include key variables in both questionnaires. The target sample size was determined in PS (Power and Sample Size Calculation Software Package, Vanderbilt University, Nashville, TN) . Based the assumption of 30% exposure among controls, we estimated that 150 cases and 300 controls were required for each study to achieve 90% power, with 95% confidence, and 5% precision to detect a variable with an odds ratio of 1.9. Details specific to each study are included below.

#### Resident study

Resident case patients were recruited from May to June 2009. Resident cases were identified both retrospectively from hospital records and prospectively for patients diagnosed during the recruitment period. A line listing of VL patients treated over the previous 12 months was compiled with the collaboration of MSF-Holland and the study team sought patients in the five most affected *kebeles* (the smallest administrative unit). Controls were selected from the two nearest neighboring houses to the case. Candidate control households were excluded and the next neighboring house selected if there was no household member meeting the age and sex matching criteria, or if there had been a case of VL in the household in the last 5 years. Candidate control individuals were screened with a focused medical history to rule out past or current VL. Those with past VL by history were excluded as controls. Persons with suspected untreated VL found in the course of fieldwork were referred to the hospital for diagnosis and treatment, and were eligible to be included as cases if confirmed.

#### Migrant study

Cases and controls for the migrant study were recruited prospectively in KAH from October 2009 to February 2011, with recruitment concentrated in the months with higher migrant influx. Migrants were defined as persons living in the area temporarily for economic purposes. Patients who met the VL case definition above and reported themselves to be migrants were eligible to participate. Controls were recruited among migrants presenting to KAH with any condition other than VL or malaria, and were recruited within a week of the corresponding case. Two age-group- and sex-matched controls were recruited for each case. Negative results by rK39 rapid test were required for inclusion. The most common diagnoses among potential controls were malaria, pneumonia, trauma and HIV-related conditions. Potential controls diagnosed with malaria (testing positive by the rapid test Paracheck Pf® [Orchid Biomedical Systems, Goa, India] for resident community controls, or by Giemsa-stained blood smear for hospital controls) were excluded.

### Data collection, management and analysis

Structured questionnaires were used to collect the data. For the resident study, trained field workers administered the questionnaire under the supervision of study team members. For the migrant study, trained hospital nurses familiar with VL diagnosis and treatment procedures administered the questionnaires. One hospital supervisor coordinated data collection in collaboration with the study team. Data were single-entered in databases designed using Epidata® software and managed by the study data manager. All questionnaires were reviewed in the field and the database and questionnaire compared by two independent observers. The data were analysed using univariable and backwards stepwise multivariable conditional regression with Wald 95% confidence limits, to account for the matched design in SAS 9.2 (SAS Institute Inc, Cary, NC). The Kruskal-Wallis test was used for comparison of continuous variables. Variables with p<0.10 in the univariable analyses was tested in multivariable model. Interaction between variables tested using interaction terms.

## Results

Each study recruited slightly more than 150 VL cases (151 residents, 157 migrants) with two matched controls per case (Table 1). Among residents, 87% of case-control sets were male, while 99% of migrant sets were male. In both studies, the mean age was early to mid-twenties. However, resident cases included 5 (3.3%) children younger than 5 years, 29 (19.2%) 5–14 years old and 13 (8.6%) adults 40 years or older, while all migrant cases except one were between 15 and 39 years old. Of the 151 resident cases, 125 (83%) were treated in the past at KAH, 24 (16%) were under treatment and 2 (1%) were new cases diagnosed during the survey. All 157 migrant cases were under treatment at the time of recruitment. Migrant cases were significantly more likely than their controls to come from Amhara regional state, whereas controls were more likely to be from Tigray (Table 1).

**Table 1:** Demographic characteristics of participants in matched case-control studies of visceral leishmaniasis among long-term residents and migrants in Humera, Tigray region, Ethiopia.

Long-term residents	Cases	Controls	P
Male	132 (87%)	264 (87%)	1.00
Female	19 (13%)	38 (13%)	
Age (years)			
Mean (median, range)	23.8 (23, 1–69)	24.7 (24.5, 1–74)	0.065
Among males			
Mean (median, range)	24.6 (24, 4–69)	25.6 (25, 1–60)	0.075
Among females			
Mean (median, range)	17.6 (16, 1–50)	18.2 (14.5, 0.8–60)	0.615
District of residence			
Kafta Humera	86 (57%)	172 (57%)	
Setit Humera	6 (4%)	12 (4%)	
T-Adiabo	54 (36%)	108 (36%)	
Sheraro	5 (3%)	10 (3%)	

Migrants	Cases	Controls	P
Male	156 (99.4)	312 (99.4)	1.0
Female	1 (0.6)	2 (0.6)	
Age (years)			
Mean (median, range)	23.6 (23, 14–38)	25.2 (24, 13–42)	0.005
Home region			
Tigray	94 (59.1%)	228 (71.7%)	–
Amhara	50 (31.5%)	62 (19.5%)	0.002
Other	15 (9.4%)	28 (8.8%)	0.37

In univariate analyses. factors associated with elevated risk in both studies included sleeping outside, on the ground or under an acacia tree (Tables 2, 3 and 4). Bed net use during the rainy season was associated with significant protection among residents (Table 3). Seasonal net use data were not collected for migrants since they were not usually in the site year-round; however, ever having used a bed net was found to be protective with the same odds ratio (0.20) as rainy season net use in residents (Table 4). Indicators of household poverty and lower educational status were associated with higher VL risk in both studies (Table 2 and 4).

**Table 2:** Resident analysis: household-level risk factors for visceral leishmaniasis, Humera, Tigray region, Ethiopia.

Factor	Cases [n/N (%)]	Controls [n/N (%)]	Matched OR^1^	95% CI	P
Owns dog	40/151 (26.5)	72/302 (23.8)	1.12	0.73–1.91	0.51
Owns any cattle (cow, bull, calf)	67/151 (44.4)	140/302 (46.4)	0.84	0.47–1.50	0.56
Owns donkey	50/151 (33.1)	103/302 (34.1)	0.94	0.565–1.551	0.80
Owns goats	51/151 (33.8)	109/302 (36.1)	0.89	0.58–1.38	0.61
Owns radio	82/151 (54.3)	178/302 (58.9)	0.82	0.55–1.23	0.33
Has latrine	84/150 (56.0)	216/302 (71.5)	0.49	0.32–0.75	0.0011
Had bed net before case kala-azar onset	62/151 (41.1)	169/300 (56.3)	0.45	0.28–0.72	0.0008
Has functional bed net now	100/130 (76.9)	229/263 (87.1)	0.50	0.27–0.90	0.0207
Earthen walls	62/151 (41.1)	169/300 (56.3)	0.45	0.28–0.72	0.0008
Walls of thatched grass on wood frame	40/151 (26.5)	41/300 (13.7)	4.45	2.14–9.28	<0.0001
Thatch roof	56/150 (37.3)	120/301 (39.9)	0.86	0.54–1.37	0.53
Earthen floor	138/150 (92.0)	282/300 (94.0)	0.74	0.32–1.69	0.47
House was ever sprayed (up to 40 years ago)	39/149 (26.1)	56/299 (18.7)	1.72	1.04–2.86	0.04
House sprayed 0–2 years before case onset	13/151 (8.6)	20/302 (6.6)	1.32	0.64–2.73	0.45
Owns land	86/151 (57.0)	177/302 (58.6)	0.92	0.59–1.43	0.71
Owns <4 hectares of land	129/151 (85.4)	264/302 (87.4)	0.84	0.48–1.49	0.56
Monthly expenditure <100 birr per person	40/151 (26.5)	36/302 (11.9)	2.81	1.66–4.76	<0.0001
Household head can write his name	89/148 (60.1)	208/301 (69.1)	0.69	0.46–1.03	0.07
Household head can read	88/150 (58.7)	207/302 (68.5)	0.66	0.44–0.98	0.04
Head of house works as laborer	41/150 (27.3)	45/302 (14.9)	2.47	1.44–4.25	0.0011
Head of house left school before class 5	124/150 (82.7)	209/302 (69.2)	2.91	1.61–5.28	0.0004
Head of household had no formal schooling	61/150 (40.7)	97/302 (32.1)	1.41	0.95–2.09	0.09
Family has 5 or more members	75/147 (51.0)	147/295 (49.8)	1.05	0.70–1.59	0.81

1 Odds ratios (OR), 95% confidence intervals (CI) and p values derived from univariable conditional logistic regression models. Number and percentage for each factor provided for reference only.

**Table 3:** Resident analysis: Behavioral risk factors for visceral leishmaniasis, Humera, Tigray region, Ethiopia.

Factor	Cases [n/N (%)]	Controls [n/N (%)]	Matched OR^1^	95% CI	P
Usually slept outside house	47/151 (31.1)	89/302 (29.2)	1.12	0.68–1.85	0.668
Usually slept on ground	42/149 (28.2)	31/300 (10.3)	4.53	2.48–8.30	<0.0001
Ever slept outside during rainy season	77/146 (52.7)	116/290 (40.0)	2.45	1.43–4.20	0.0011
Ever slept outside during dry season	136/145 (93.8)	264/292 (90.4)	1.76	0.77–4.03	0.183
Always slept outside during rainy season	39/146 (26.7)	44/290 (15.2)	2.41	1.39–4.18	0.0018
Always slept outside during dry season	84/145 (57.9)	182/292 (62.3)	0.81	0.50–1.31	0.39
Always slept under net in rainy season	57/143 (39.9)	188/241 (78.0)	0.20	0.12–0.34	<0.0001
Always slept under net in dry season	12/143 (8.4)	32/241 (13.3)	0.67	0.31–1.43	0.297
Slept under acacia at night	40/143 (28.0)	36/283 (12.7)	6.40	2.77–14.79	<0.0001
Slept under acacia during day	76/142 (53.5)	85/288 (29.5)	4.07	2.32–7.16	<0.0001

1 Odds ratio (OR), 95% confidence intervals (CI) and p values derived from univariable conditional logistic regression models. Number and percentage for each factor provided for reference only.

**Table 4:** Migrant analysis: risk factors for visceral leishmaniasis in Humera, Tigray region, Ethiopia.

Factor	Cases [n/N (%)]	Controls [n/N (%)]	Matched OR^1^	95% CI	P
HIV infection	12/146 (8.2)	8/276 (2.8)	4.3	1.5–12.8	0.008
Home is Amhara Region (compared to other regions)	50/159 (31.5)	62/318 (19.5)	2.0	1.28–3.24	0.003
Living in Humera for 1 year or more	102/156 (65.4)	157/309 (50.8)	1.8	1.2–2.7	0.005
Presence of cattle in workplace	33/155 (21.3)	50/297 (16.8)	1.4	0.8–2.4	0.22
Presence of donkey in workplace	20/153 (13.1)	41/295 (13.9)	0.95	0.5–1.8	0.87
Presence of goats in workplace	24/156 (15.4)	40/299 (13.4)	1.2	0.7–2.2	0.54
Presence of dogs in workplace	31/154 (20.1)	43/295 (14.6)	1.6	0.9–2.7	0.11
Slept near cattle	19/153 (12.4)	10/310 (3.2)	3.7	1.7–8.0	0.0008
Slept near donkey	9/154 (5.8)	6/308 (2.0)	3.0	1.1–8.4	0.04
Slept near goats	11/153 (7.2)	8/308 (2.6)	2.9	1.1–7.4	0.04
Slept near dogs	13/152 (8.6)	8/307 (2.6)	3.5	1.4–8.9	0.008
Termite mound within 5 meters of sleeping place	36/136 (26.5)	35/230 (15.2)	1.8	1.1–3.3	0.04
Eats meat at least once per month	59/157 (37.6)	180/312 (57.7)	0.38	0.25–0.60	<0.0001
Eats porridge as staple (rather than injera)	110/157 (70.1)	106/312 (34.0)	6.7	4.0–11.2	<0.0001
Left school before class 5	113/159 (71.1)	177/317 (55.8)	2.12	1.36–3.31	0.0008
No formal schooling	83/159 (52.2)	84/317 (26.5)	3.5	2.2–5.4	<0.0001
Four or more people share sleeping place	73/153 (47.7)	156/309 (50.5)	0.88	0.59–1.3	0.54
Usually slept outside house in dry season	64/143 (44.8)	78/307 (25.4)	2.6	1.7–4.1	<0.0001
Usually slept outside house in wet season	63/144 (43.8)	78/304 (25.7)	2.7	1.6–4.4	0.0001
Usually slept on ground in dry season	71/157 (45.2)	92/314 (29.3)	2.3	1.4–3.5	0.0003
Usually slept on ground in wet season	89/157 (56.7)	123/314 (39.2)	2.5	1.6–4.0	<0.0001
Ever uses bed net	82/155 (52.9)	254/308 (82.5)	0.20	0.11–0.33	<0.0001
Slept under acacia at night	42/156 (26.9)	16/313 (5.1)	6.9	3.5–13.5	<0.0001
Slept under acacia during day	76/157 (48.4)	53/313 (16.9)	6.2	3.6–10.7	<0.0001

1 Odds ratios (OR), 95% confidence intervals (CI) and p values derived from univariable conditional logistic regression models. Number and percentage for each factor provided for reference only.

Some significant risk factors were found only in one of the studies. In the resident study, having house walls constructed from grass thatch was associated with an odds ratio of 4.5 compared to walls made from earth or other materials (Table 2). Data on HIV status were available only in the migrant study; HIV infection was associated with a 4-fold increase in VL risk (Table 4). Increased risk was also seen for migrants who slept near cattle, dogs or termite mounds, ate meat less than once per month, and those that ate porridge rather than injera as a staple food. Risk was higher for migrants who had spent a year or more in Humera compared to those with shorter duration of exposure there, but higher for those coming from Amhara than from Tigray, where Humera is located.

**Figure 2:**
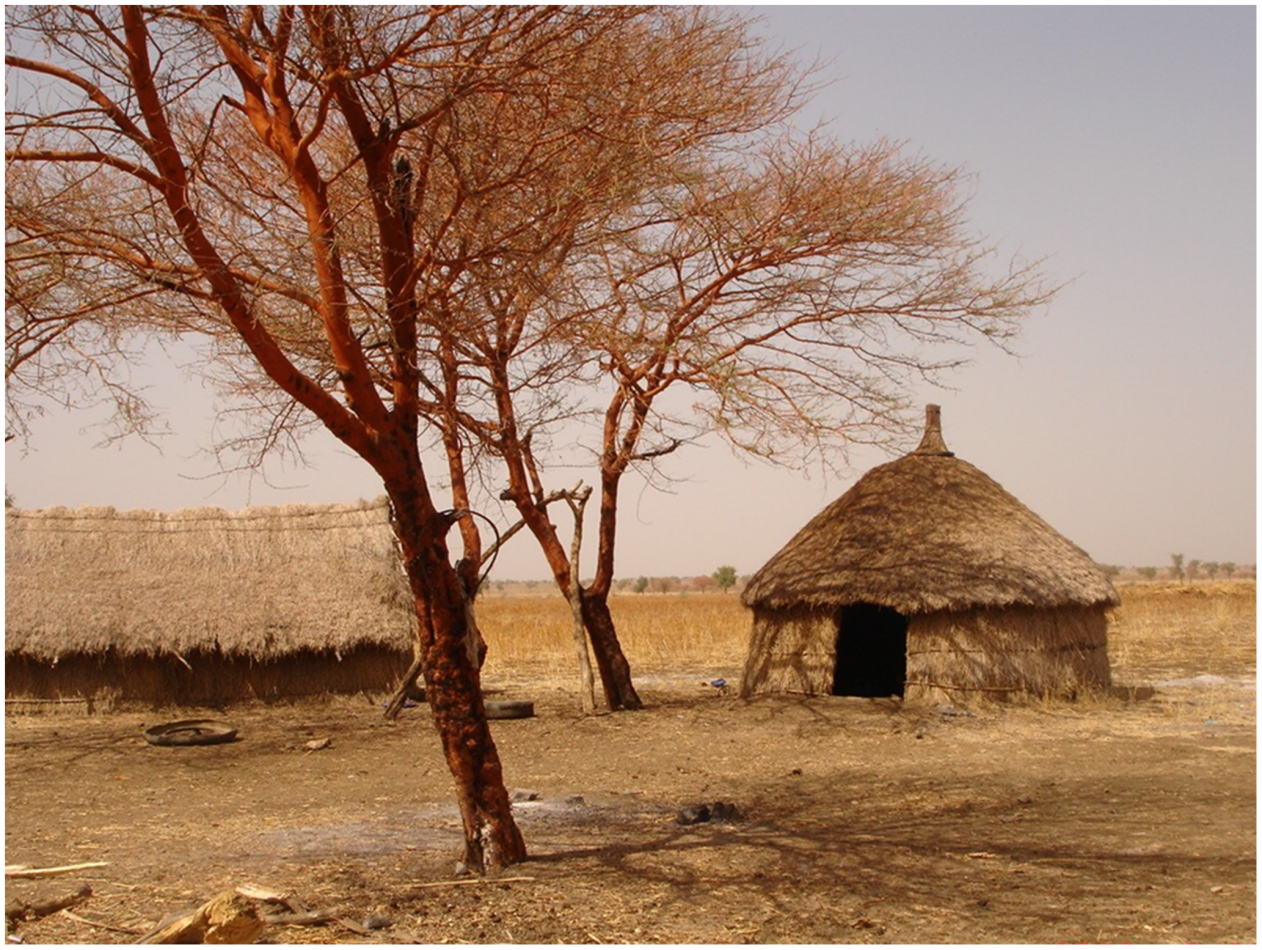
Typical Humera shelter with walls of grass thatch and nearby acacia tree.

In multivariable conditional regression models, both datasets demonstrated elevated risk associated with sleeping under an acacia tree at night (odds ratio (OR) 5.22 [95% confidence interval 1.66–16.42] and 4.74 [1.88–11.99] for residents and migrants respectively), indicators of poverty (OR 3.22 [1.42–7.33] for low income for residents, OR 4.65 [2.33–9.29] for use of porridge as the staple among migrants) and lower educational status (Table 5). Strong protective effects were observed in both studies for bed net use (OR 0.24 [0.12–0.48] for use in the rainy season for residents, 0.20 [0.10–0.42] for ever used net among migrants). For residents, living in a house with thatch walls (Figure 2) rather than earth conferred 5-fold and sleeping on the ground close to 3-fold increased risk. Among migrants, the risk associated with HIV status was borderline significant and sleeping near dogs was associated with 7-fold increased risk.

**Table 5:** Multivariable conditional logistic regression models for factors associated with risk of visceral leishmaniasis among residents and migrants in Humera, Tigray, Ethiopia.

Factor	Matched OR^1^	95% CI^1^	P
**Residents**			
Always slept under net in rainy season	0.24	0.12–0.48	<.0001
Slept under acacia at night	5.22	1.66–16.42	0.005
Usually slept on ground	2.96	1.20–7.30	0.02
Walls of thatched grass on wood frame	5.30	1.34–21.03	0.02
Monthly expenditure <100 birr per person	3.22	1.42–7.33	0.005
Head of house left school before class 5	2.78	1.17–6.59	0.02
**Migrants**			
HIV infection^2^	3.98	0.94–16.9	0.06
Ever slept under net	0.20	0.10–0.42	<0.0001
Slept under an acacia at night	4.74	1.88–11.99	0.001
Slept near dogs	6.79	1.83–25.16	0.004
Staple food is porridge (rather than injera)	4.65	2.33–9.29	<0.0001
No formal schooling	5.02	2.59–9.74	<0.0001

1 Odds ratios (OR) and 95% confidence intervals (CI).

2 Data on HIV status missing for at least one member of 21 case-control sets; these sets therefore excluded from analysis.

## Discussion

Although the northern lowland border with Sudan has long been known as the most important VL-endemic zone in Ethiopia, our data represent the first systematic evaluations of risk factors for disease in the area. Several overarching themes emerge from both the resident and migrant data. Poverty and low educational status are strong underlying factors in both studies. Environmental associations also emerged, including sleeping under acacia trees and in more exposed settings (in houses with thatch walls, outside or on the ground). A protective effect of bed net use was seen in both studies, and supports the policy of net use as a control strategy. The massive scale-up of insecticide-treated nets for malaria control launched by the Ministry of Health in 2005 may therefore have collateral benefits for VL control [15].

Humera is located near the Eritrean and Sudanese borders, acting as a node for cross-border trade and traffic. An estimated 250,000 migrant workers come to the area each farming season (personal communication, Kafta-Humera District Health Office, 2010). Many migrants come from the highlands, where until recently there was no reported VL transmission. Return of newly infected migrant workers was hypothesized to have triggered the VL epidemic in Libo Kem Kem and Fogera in the mid-2000s [2]. Even now, transmission is focal and uncommon compared to the lowland areas, so most highland residents are likely to have no pre-existing immunity to VL. Population mobility and socioeconomic vulnerability also contribute to HIV transmission in the border region, where VL is a major opportunistic infection. Several years prior to our study, the reported prevalence was 11–13% in counseling and testing centers and 29% among VL patients [13]. In 2008, KAH treated a total of 376 VL patients of whom 39% were migrant workers; 23% of migrants and 40% of residents were HIV positive (MSF-H, unpublished data). The HIV prevalence seen in our migrant VL cases was substantially lower than in previous reports, reflecting a parallel decrease in HIV prevalence at national level and at KAH (2010, 7.5%; 2011, 6.5%; 2012, 8.2%), as well as the decentralization of ART services in the region.

Risk associated with nocturnal outdoor exposure was also seen in our earlier study in Libo Kem Kem [16]. In the current study, the constellation of factors associated with elevated risk suggests that sand fly feeding occurred predominantly outside of houses, or in thatch-walled houses that are highly vulnerable to sand fly entry and resting. Based on sand fly trapping data, *P. orientalis* has generally been considered exophilic and exophagic [8], [9]. However, in a study in villages with apparent domestic VL transmission in eastern Sudan, 88% of flies were trapped inside houses or grain storerooms, raising the possibility that feeding may occur outdoors or indoors depending on local housing and environmental conditions [9], [17]. In our study, sleeping under an acacia tree during the day or at night conferred 4- to 7-fold increased risk of VL. Acacia trees appear to have a special relationship with *P. orientalis*, with high sand fly densities found in association with acacia-balanites forest [8], [9]. Experts hypothesize that this may be due to the microclimate around acacia thickets and/or the presence of specific sugar meal sources such as the balanites fruit or the secretions of aphids and coccids found in these settings [9].

We found no association with animal ownership in our resident analysis, in contrast to findings in Libo where dog ownership was linked to increased risk and Sudan where both dogs and cattle appeared as risk factors for VL [16], [18]. In univariate analyses, we found increased risk for migrants who reported sleeping near cattle, in contrast to the protective association found for proximity to cattle in studies in Kenya and Bangladesh [19], [20]. Nevertheless, in the migrant multivariable model, there was no significant risk associated with sleeping near cattle while sleeping near dogs was associated with nearly 7-fold increased risk. Whether this represents a direct risk from infected dogs, or whether this variable represents a proxy for other outdoor exposures such as increased vector density, is unclear. Although transmission is thought to be predominantly anthroponotic in these settings, infected dogs have been found by serology or PCR in several studies in Ethiopia as well as Sudan [2], [3], [21]. However, the role of the dog as an epidemiologically important infection reservoir host in this area is not yet clear.

The two studies we performed used different recruitment methods for logistical reasons, and this difference may have affected characteristics of the control groups, in particular. The use of village controls, as in the resident study, is generally considered optimal in terms of comparability of case and control groups. We chose resident controls with no history of kala-azar in the household in the previous 5 years, because of the strong household clustering seen in VL, the fact that many of the factors evaluated are household-level variables, and the high likelihood that household members of kala-azar cases might have undetected subclinical VL infection [19], [22], [23]. Thus, inclusion of controls from affected households could bias results for household-level variables toward the null hypothesis. At the same time, exclusion of these households could lead to a control group with lower likelihood of VL and bias results for individual-level variables in the opposite direction. A solution to this dilemma would be to perform serology and leishmanin skin testing on all potential controls, allowing separate analyses of VL disease and infection [22]. However, the requirement for extra manpower, resources and time precluded this option. There was no practical way to recruit appropriate community controls for the migrant study. Most migrants were sleeping in temporary shelters that moved along with the farm work. Although the use of hospital-based controls may have introduced some biases with respect to exposures, these factors could bias either toward the null hypothesis (e.g. if those who are ill are more likely to share risk factors with the case group) or away from it (if ill persons are more likely to use personal protective measures). Our inability to include existing HIV data in the resident analysis due to ethics committee-imposed restrictions constituted a further limitation. Nevertheless, the consistency of our findings between the two studies, as well as their agreement with many of the risk factors expected based on the known transmission characteristics of VL in this region, support the validity of our findings.

Finally, in our studies as in many others, VL risk was strongly associated with poverty and factors linked to poverty [24]. Although the details of the variables differed between residents and migrants, low household income, poorer dietary options and lower educational attainment were all strongly associated with higher risk. The dietary variables in the migrant study are of particular interest. Ethiopians, especially those from the highlands, prefer injera over other staple foods; eating porridge made from sorghum was associated with almost 5-fold increased risk of VL, but it is unclear whether this was due to lower nutritional value or simply because eating porridge is a potent indicator of poverty. Eating meat at least once per month was associated with significantly lower risk, possibly because, in the setting of nutritionally impoverished diets, even meager consumption of meat translates into slightly better micronutrient status and lower risk of progression to clinical disease [22]. Although VL rapid tests and treatment drugs are provided free of charge, other hospital costs are not. Health care access is impeded for migrant workers by the need for a supporting letter from their administrative office of origin in order to access free services from the hospital. This leads to delays in diagnosis and treatment with poor outcomes. The extreme vulnerability, especially of migrant workers, must be taken into account when designing and implementing control strategies. For example, strategies that depend on house spraying will have little impact when a significant proportion of transmission is outdoors and many migrants have no fixed abode. A better approach might be innovative bed net deployment that makes net use practical for those sleeping outside and moving from place to place [25]. Programs to protect migrant workers from VL will yield additional dividends if future introductions of the disease into non-endemic areas can be prevented.

## Supporting Information

Checklist S1 STROBE checklist.
(DOCX)
